# Representation of navigational affordances and ego-motion in the occipital place area

**DOI:** 10.1162/imag_a_00424

**Published:** 2025-01-10

**Authors:** Frederik S. Kamps, Emily M. Chen, Nancy Kanwisher, Rebecca Saxe

**Affiliations:** Department of Brain and Cognitive Sciences, Massachusetts Institute of Technology, Cambridge, MA, United States; Department of Psychology, School of Philosophy, Psychology, and Language Sciences, University of Edinburgh, Edinburgh, United Kingdom

**Keywords:** scene perception, vision, navigation, affordances, functional magnetic resonance imaging (fMRI), occipital place area

## Abstract

Humans effortlessly use vision to plan and guide navigation through the local environment, or “scene.” A network of three cortical regions responds selectively to visual scene information, including the occipital (OPA), parahippocampal (PPA), and medial place areas (MPA)—but how this network supports visually guided navigation is unclear. Recent evidence suggests that one region, in particular, the OPA, supports visual representations for navigation, while PPA and MPA support other aspects of scene processing. However, most previous studies tested only static scene images, which lack the dynamic experience of navigating through scenes. We used dynamic movie stimuli to test whether OPA, PPA, and MPA represent two critical kinds of navigationally relevant information: navigational affordances (e.g., can I walk to the left, right, or both?) and ego-motion (e.g., am I walking forward or backward? turning left or right?). We found that OPA is sensitive to both affordances and ego-motion, as well as the conflict between these cues—for example, turning toward vs. away from an open doorway. These effects were significantly weaker or absent in PPA and MPA. Responses in OPA were also dissociable from those in early visual cortex, consistent with the idea that OPA responses are not merely explained by lower-level visual features. OPA responses to affordances and ego-motion were stronger in the contralateral than in ipsilateral visual field, suggesting that OPA encodes navigationally relevant information within an egocentric reference frame. Taken together, these results support the hypothesis that OPA contains visual representations that are useful for planning and guiding navigation through scenes.

## 1 Introduction

**Fig. 1.**
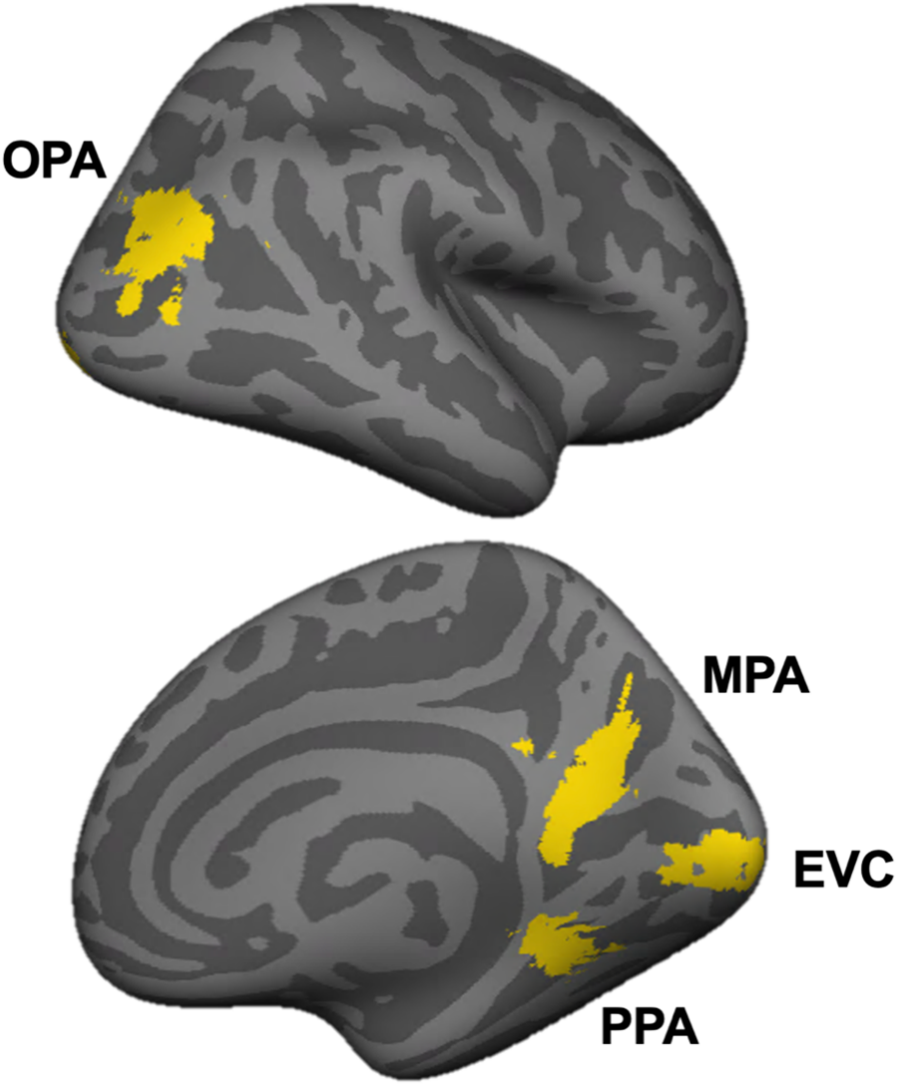
Regions of interest. Regions of interest (ROIs) were functionally defined in individual subjects using independent localizer data. For scene-selective regions (OPA, PPA, and MPA), an initial region of interest was selected manually based on the contrast of scenes greater than objects, and then the top 50 voxels (in each hemisphere, using the same contrast) were selected from this initial, manually defined region as the final ROI. For early visual cortex (EVC), an initial region of interest was defined using an anatomical mask, and then the top 50 voxels responding to all conditions minus fixation (in each hemisphere) were selected as the final ROI. For visualization purposes here, the masks for each ROI in each subject were summed across subjects and binarized, revealing the spatial distribution of selected voxels for each region. ROIs were defined and analyzed bilaterally, but are shown in the right hemisphere only here.

Humans expertly use vision to plan and guide navigation through scenes. The past several decades have revealed a network of at least three cortical regions that respond selectively to visual scene information, including the occipital place area (OPA) (Dilks et al., 2013), parahippocampal place area (PPA) (Epstein & Kanwisher, 1998), and medial place area (MPA; also known as retrosplenial complex) (Maguire, 2001;Silson et al., 2016) (Fig. 1). It has recently been hypothesized that these regions are functionally dissociated, with PPA supporting scene categorization (e.g., am I in a kitchen or a forest?), MPA supporting memory-guided navigation (e.g., which way should I head to find my hotel, six blocks away?), and OPA supporting visually guided navigation (e.g., can I walk to the left or the right?) (Dilks et al., 2022).

Of the three scene-selective regions, the least is known about OPA (Julian et al., 2012). Nevertheless, growing evidence supports the hypothesis that OPA is specialized for visually guided navigation (Dilks et al, 2011;Kamps et al., 2016a;Persichetti & Dilks, 2016). For example, OPA responds significantly more when participants are asked to indicate how they would navigate through a scene than when asked to indicate the category of the same scene (PPA showed the opposite pattern, responding more to the categorization task than the navigation task; MPA showed no preference) (Persichetti & Dilks, 2018). Two further studies found that OPA (but not PPA or MPA) is sensitive to navigational affordances (i.e., how the spatial structure of the local environment constrains where one can or cannot locomote). For example, patterns of activation in OPA, in response to static snapshots of novel scenes, contain reliable information about the location of open doorways that afford navigation to rooms beyond the current view (Bonner & Epstein, 2017), and the presence of spatial boundaries that limit locomotion (Park & Park, 2020). OPA is also sensitive to the perceived distance (Persichetti & Dilks, 2016) and direction (Dilks et al., 2011) of scene information, suggesting an ego-centric frame of reference, which is critical for planning and guiding one’s own movements through space.

Most studies to date (including all studies cited above) have relied on static images to study OPA responses. Yet in real life, navigation is inherently dynamic: visual scenes are seen in motion as the observer moves through them (i.e., ego-motion). This ego-motion generates patterns of optic flow that can be used to support essential aspects of navigation, such as judging the current heading with respect to the scene, or time-to-contact for obstacles and boundaries in the space (Gibson, 1979). Accordingly, if OPA is specialized for visually guided navigation, then the full functional profile of OPA may only be revealed during dynamic, first-person motion through scenes. Consistent with this possibility, five studies suggest that OPA is sensitive to dynamic scene information (but seeKennedy et al., 2024).Kamps et al. (2016b),Kamps et al. (2020), andSuzuki et al. (2021)showed that OPA responds significantly more to dynamic scenes depicting first-person perspective motion through scenes than to static images taken from these same scene videos (with no such motion enhancement found for faces or objects). Similarly,Hacialihafiz et al. (2015)showed that OPA responds significantly more to dynamic scene displays depicting linear horizontal motion (e.g., panning across a scene image from left to right) than static versions of those same displays. Finally,Sulpizio et al. (2020)found that OPA responds more to point-light displays depicting ego-motion-compatible optic flow than random motion. For all five studies, responses to motion information in OPA were greater than those in PPA or MPA, providing initial evidence for a functional dissociation in the scene network based on motion sensitivity. Taken together, these studies demonstrate the robustness of OPA’s sensitivity to ego-motion information, even across the very different stimuli used to interrogate ego-motion representation (e.g., including minimal point-light stimuli, computer-generated virtual scenes, and videos of ego-motion through real scenes). However, none of these studies has explored the finer grained information that OPA extracts from dynamic scenes. It is, therefore, unclear to what extent OPA responses to dynamic scenes reflect information processing relevant to planning and guiding navigation.

**Fig. 2.**
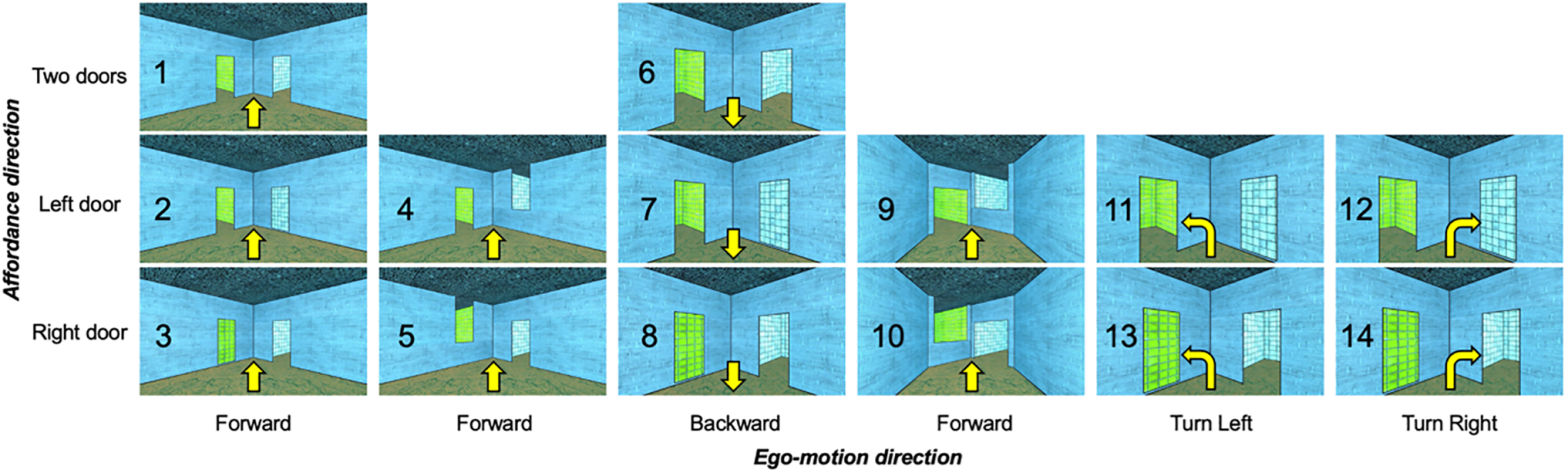
Dynamic movie stimuli. Adult participants viewed 3 s video clips in an event-related design. The still images shown here depict the first frame of each movie stimulus. Condition numbers are listed over each stimulus. The direction of navigational affordance information (i.e., open doorways) in each scene is organized by row, depicting either two affordances (top row), an affordance to the left, but not right (middle row), or an affordance to the right, but not the left (bottom row). The direction of ego-motion is organized by column, and further indicated by the yellow arrow over each image, depicting either forward (first column), backward (second column), left turn (third column), or right turn (fourth column) ego-motion.

To better understand the nature of dynamic scene representations in OPA, we tested whether OPA extracts two kinds of navigationally relevant information in dynamic scenes: (1)*navigational affordances*(is there an open doorway to the left, right, or both?) and (2)*ego-motion directions*(moving forward or backward, turning left or right), as well as the conflict of these two kinds of information (e.g., turning toward vs. away from an open doorway) (Fig. 2). We predicted that OPA represents both navigational affordances and ego-motion directions, as well as their conflict, and that responses to such navigationally relevant information would be stronger in OPA than in both PPA and MPA, consistent with the hypothesized functional dissociations between these regions. An important further question concerns the extent to which responses to navigationally relevant information in OPA could reflect low-level, retinotopic properties of the stimuli that are confounded with navigationally relevant information (e.g., left turn stimuli will generate greater motion energy in the right visual field, compared with the left visual field), rather than more abstract, higher-level navigationally relevant invariances (Groen et al., 2017). To address this question, we also compared responses in OPA with those in early visual cortex (EVC). EVC has robust retinotopic spatial structure and is highly sensitive to motion energy, but unlike OPA, EVC is not thought to support higher-level navigational processing, and, therefore, provides a proxy for lower-level, retinotopic processing. If OPA represents higher-level visual information relevant for navigation, then responses to navigationally relevant information in OPA will differ from those in EVC. The results confirmed both sets of predictions. Note though that the analyses reported here include minor deviations from preregistered analysis plan. Results from the originally planned analyses are described in theSupplemental Materials.

## 2 Methods

### 2.1 Participants

Sixteen participants (mean age = 29.3 years, range = 20–45 years; 9 females) were recruited from the MIT community. All participants gave written informed consent to participate and had normal or corrected-to-normal vision. The sample size was preregistered prior to analysis, and was selected on the basis of prior fMRI studies testing representation of navigational affordances in OPA (Bonner & Epstein, 2017;Park & Park, 2020). A power analysis for the critical paired-samples t-tests suggested that a sample size of 16 provided 85% power to detect large effects (i.e., d = 0.8; if we assumed the even larger effect sizes reported in previous work (e.g., d = 1.02 inBonner and Epstein (2017), power increased to 97%). Recruitment and experiment protocols were approved by the Committee on the Use of Humans as Experimental Subjects (COUHES) at the Massachusetts Institute of Technology.

### 2.2 Design

We used a region of interest (ROI) approach in which we used one set of runs (“Localizer Runs”) to localize ROIs based on functional signatures, and a distinct independent set of runs (“Experimental Runs”) to investigate responses of these regions to the Experimental conditions shown inFigure 2, using both univariate and multivariate approaches (for a detailed description of this analysis, see Data analysis section below).

Localizer stimuli consisted of 3 s videos of dynamic Scenes, Objects, Faces, and Scrambled Objects, as described previously inKamps et al. (2016b)andKamps et al. (2020). Stimuli were presented using a block design at 13.7 x 18.1 degrees of visual angle. Each run was 315 s long and contained 4 blocks per stimulus category. The order of the first set of blocks was pseudorandomized across runs (e.g., Faces, Objects, Scenes, Scrambled) and the order of the second set of blocks was the palindrome of the first (e.g., Scrambled, Scenes, Objects, Faces). Each block consisted of 5 2.8 s video clips from a single condition, with an ISI of 0.2 s, resulting in 15 s blocks. Each run also included five fixation blocks: one at the beginning, three evenly spaced throughout the run, and one at the end. Participants completed three localizer runs, interleaved between every two experimental runs.

**Fig. 3.**
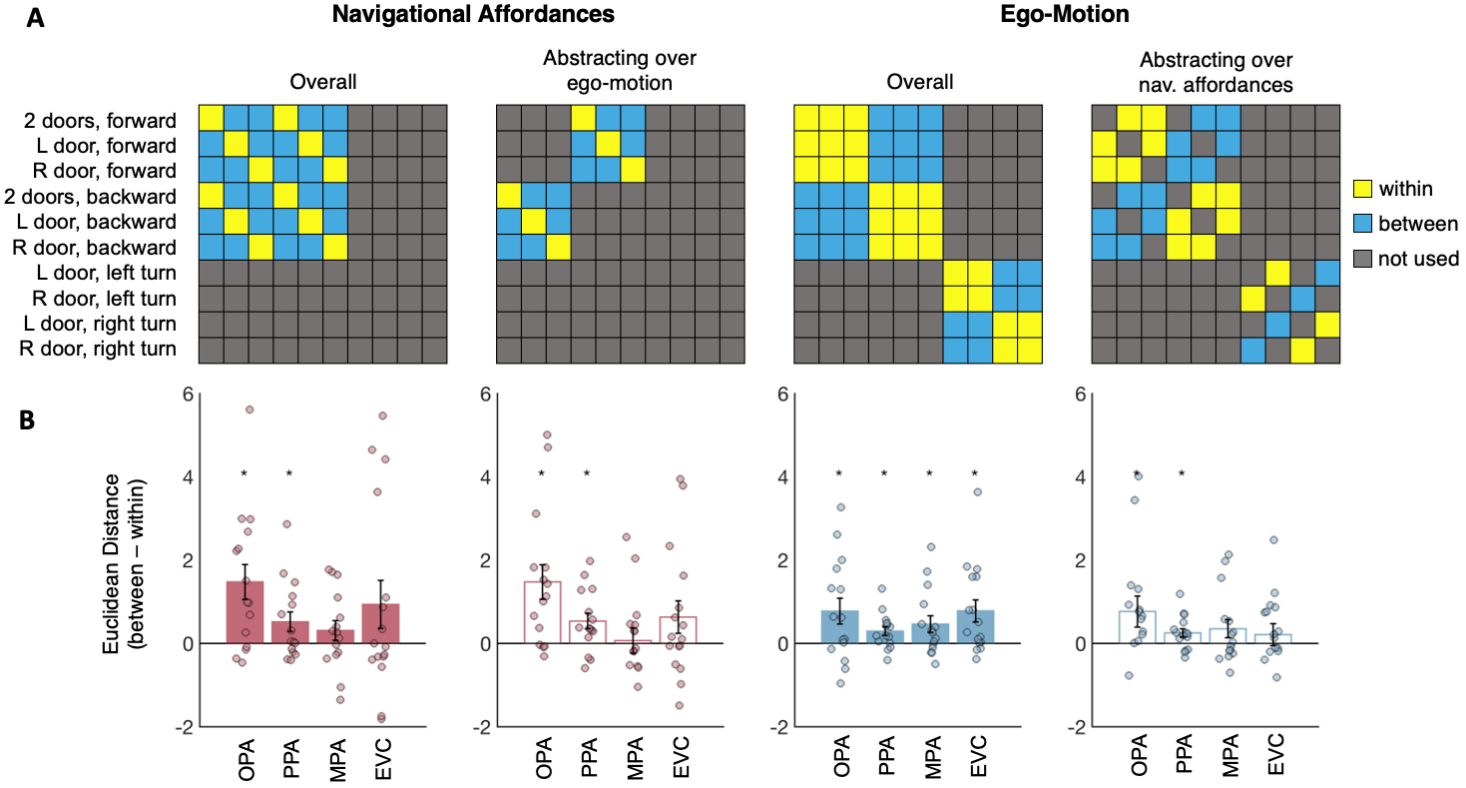
Multivariate analyses of navigational affordance and ego-motion representation*.*(A) Hypothesis matrices used to test navigational affordance and ego-motion representation. If OPA represents navigational affordances (left-most matrix) and/or ego-motion (second-from-the-right matrix), then voxel-wise patterns of activity will be more similar (i.e., smaller Euclidean distance) between conditions with the same navigational affordance or ego-motion information (yellow cells) than conditions with different information (blue cells). Navigational affordance and ego-motion information were tested “overall” (solid bars in bar plots, bottom row), as well as generalizing across the other kind of information (empty bars; e.g., testing navigational affordance only across conditions that differ in ego-motion, and vice versa for ego-motion information, generalizing across navigational affordances). (B) Results of the four multivariate decoding analyses in the occipital place area (OPA), parahippocampal place area (PPA), medial place area (MPA), and an early visual cortex (EVC) control region. Bar plots indicate the difference score for the “between” conditions minus the “within” conditions; responses greater than zero indicate more similar responses (i.e., smaller Euclidean distance) for the “within”- than “between”-condition pairs, and thus significant decoding. AU indicates arbitrary units of Euclidean distance in fMRI response space. Error bars represent the standard error of the mean. Markers depict data from individual participants. Asterisks indicate p < 0.05, based on paired samples t tests comparing between vs. within conditions. Observed neural representational similarity matrices for each region are shown inSupplemental Figure 1.

Experimental stimuli consisted of 14 conditions (Conditions 1–10 are shown inFigure 2; example stimuli and results from conditions 11–14 are shown inSupplemental Fig. 3). All stimuli were created using Unity software and depicted 3 s clips of the first-person experience of walking through scenes. Navigational affordances were manipulated by including an open doorway to either the left side, right side, or both sides. To help control for low-level visual confounds, the non-doorway side always included a distractor object, either a painting (conditions 1–10) or an inverted doorway (conditions 11–14). Furthermore, the textures applied to the painting and the walls through the doorways were counterbalanced, such that each texture appeared equally on either side across the full stimulus set. Ego-motion was manipulated by changing the direction of ego-motion through scene, which could either be forward (conditions 1–3, 11–14), backward (conditions 4–6), a left turn (conditions 7–8), or a right turn (conditions 9–10). To help prevent visual adaptation over the course of the experiment, the 14 experimental conditions were counterbalanced across 8 room types, which differed from one another based on the textures applied to the walls, floor, and ceiling, and to a lesser extent, by the size and shape of the doorways and corresponding distractor (Fig. 5). Stimuli were presented at 13.1 x 18.6 DVA in an event-related paradigm. Each stimulus was presented for 2.5 s, followed by a minimum inter-stimulus interval (ISI) of 3.5 s and a maximum ISI of 9.5 s, optimized separately for each run using OptSeq2. Participants viewed 4 repetitions of each condition per run, and completed 8 experimental runs, yielding 32 total repetitions per condition across the experiment. To help ensure participants paid attention throughout the experiment, participants performed a one-back task, responding via button press whenever the exact same video stimulus repeated on back-to-back trials. Participants were also instructed to lie still, keep their eyes open, and try to “pay attention to” and “immerse themselves in” the stimuli.

### 2.3 Data acquisition

Data were acquired from a 3-Tesla Siemens Magnetom Prisma scanner located at the Athinoula A. Martinos Imaging Center at MIT, using a 32-channel head coil. Participants viewed movie stimuli through a mirror projected to a screen behind the scanner. Scout images (3D low-resolution anatomical scans) were acquired using auto-align in 128 sagittal slices with 1.6 mm isotropic voxels (TA = 0.14; TR = 3.15 ms; FOV = 260 mm). Anatomical T1-weighted structural images were acquired in 176 interleaved sagittal slices with 1.0 mm isotropic voxels (MPRAGE; TA = 5:53; TR = 2530.0 ms; FOV = 256 mm; GRAPPA parallel imaging, acceleration factor of 2). Functional data were acquired with a gradient-echo EPI sequence sensitive to Blood Oxygenation Level Dependent (BOLD) contrast in 2 mm isotropic voxels in 46 interleaved near-axial slices covering the whole brain (EPI factor = 70; TR = 2 s; TE = 30.0 ms; flip angle = 90 degrees; FOV = 210 mm). For the localizer runs, 158 volumes were acquired per run (TA = 5:16). For the experimental runs, 228 volumes were acquired per run (TA = 7:36). Due to experimenter and technical errors, the actual number of volumes collected occasionally deviated from these values. In these cases, all timepoints were included in the analysis, with only usable trials included in the GLM.

### 2.4 Preprocessing

Results included in this manuscript come from preprocessing performed using fMRIPrep 21.0.1 (Esteban et al., 2019, RRID:SCR_016216), which is based on Nipype 1.6.1 (K. Gorgolewski et al., 2011, RRID:SCR_002502).

#### 2.4.1 Anatomical data preprocessing

One T1-weighted (T1w) image was collected per participant. The T1-weighted (T1w) image was corrected for intensity non-uniformity (INU) with N4BiasFieldCorrection (Tustison et al., 2010), distributed with ANTs 2.3.3 (Avants et al., 2008, RRID:SCR_004757), and used as T1w-reference throughout the workflow. The T1w-reference was then skull-stripped with a Nipype implementation of the antsBrainExtraction.sh workflow (from ANTs), using OASIS30ANTs as target template. Brain tissue segmentation of cerebrospinal fluid (CSF), white matter (WM), and gray matter (GM) was performed on the brain-extracted T1w using fast (FSL 6.0.5.1:57b01774, RRID:SCR_002823,Zhang et al., 2001). Brain surfaces were reconstructed using recon-all (FreeSurfer 6.0.1, RRID:SCR_001847,Dale et al., 1999), and the brain mask estimated previously was refined with a custom variation of the method to reconcile ANTs-derived and FreeSurfer-derived segmentations of the cortical gray matter of Mindboggle (RRID:SCR_002438,Klein et al., 2017). Volume-based spatial normalization to two standard spaces (MNI152NLin6Asym, MNI152NLin2009cAsym) was performed through nonlinear registration with antsRegistration (ANTs 2.3.3), using brain-extracted versions of both T1w reference and the T1w template. The following templates were selected for spatial normalization: FSL\u2019s MNI ICBM 152 nonlinear 6th Generation Asymmetric Average Brain Stereotaxic Registration Model [Evans et al. (2012), RRID:SCR_002823; TemplateFlow ID: MNI152NLin6Asym], ICBM 152 Nonlinear Asymmetrical template version 2009c [Fonov et al. (2009), RRID:SCR_008796; TemplateFlow ID: MNI152NLin2009cAsym].

#### 2.4.2 Functional data preprocessing

For each of the 9–12 BOLD runs per subject (across all tasks and sessions), the following preprocessing was performed. First, a reference volume and its skull-stripped version were generated using a custom methodology of fMRIPrep. Head-motion parameters with respect to the BOLD reference (transformation matrices, and six corresponding rotation and translation parameters) are estimated before any spatiotemporal filtering using mcflirt (FSL 6.0.5.1:57b01774,Jenkinson et al., 2002). The BOLD time series (including slice-timing correction when applied) were resampled onto their original, native space by applying the transforms to correct for head-motion. These resampled BOLD time series will be referred to as preprocessed BOLD in original space, or just preprocessed BOLD. The BOLD reference was then co-registered to the T1w reference using bbregister (FreeSurfer) which implements boundary-based registration (Greve & Fischl, 2009). Co-registration was configured with six degrees of freedom. Several confounding time series were calculated based on the preprocessed BOLD: frame-wise displacement (FD), DVARS, and three region-wise global signals. FD was computed using two formulations following Power (absolute sum of relative motions,Power et al. (2014)) and Jenkinson (relative root mean square displacement between affines,Jenkinson et al. (2002)). FD and DVARS are calculated for each functional run, both using their implementations in Nipype (following the definitions byPower et al. (2014)). The three global signals are extracted within the CSF, the WM, and the whole-brain masks. Additionally, a set of physiological regressors were extracted to allow for component-based noise correction (CompCor,Behzadi et al., 2007). Principal components are estimated after high-pass filtering the preprocessed BOLD time series (using a discrete cosine filter with 128 s cutoff) for the two CompCor variants: temporal (tCompCor) and anatomical (aCompCor). tCompCor components are then calculated from the top 2% variable voxels within the brain mask. For aCompCor, three probabilistic masks (CSF, WM, and combined CSF+WM) are generated in anatomical space. The implementation differs from that of Behzadi et al. in that instead of eroding the masks by two pixels on BOLD space, the aCompCor masks are subtracted a mask of pixels that likely contain a volume fraction of GM. This mask is obtained by dilating a GM mask extracted from the FreeSurfer (2019) aseg segmentation, and it ensures components are not extracted from voxels containing a minimal fraction of GM. Finally, these masks are resampled into BOLD space and binarized by thresholding at 0.99 (as in the original implementation). Components are also calculated separately within the WM and CSF masks. For each CompCor decomposition, the k components with the largest singular values are retained, such that the retained components\u2019 time series are sufficient to explain 50% of variance across the nuisance mask (CSF, WM, combined, or temporal). The remaining components are dropped from consideration. The head-motion estimates calculated in the correction step were also placed within the corresponding confounds file. The confound time series derived from head-motion estimates and global signals were expanded with the inclusion of temporal derivatives and quadratic terms for each (Satterthwaite et al., 2013). Frames that exceeded a threshold of 0.5 mm FD or 1.5 standardized DVARS were annotated as motion outliers. The BOLD time series were resampled into standard space, generating a preprocessed BOLD run in MNI152NLin6Asym space. First, a reference volume and its skull-stripped version were generated using a custom methodology of fMRIPrep. Automatic removal of motion artifacts using independent component analysis (ICA-AROMA,Pruim et al., 2015) was performed on the preprocessed BOLD on MNI space time series after removal of non-steady-state volumes and spatial smoothing with an isotropic, Gaussian kernel of 6 mm FWHM (full-width half-maximum). Corresponding non-aggressively denoised runs were produced after such smoothing. Additionally, the aggressive noise regressors were collected and placed in the corresponding confounds file. All resamplings can be performed with a single interpolation step by composing all the pertinent transformations (i.e. head-motion transform matrices, susceptibility distortion correction when available, and co-registrations to anatomical and output spaces). Gridded (volumetric) resamplings were performed using antsApplyTransforms (ANTs), configured with Lanczos interpolation to minimize the smoothing effects of other kernels (Lanczos, 1964). Non-gridded (surface) resamplings were performed using mri_vol2surf (FreeSurfer).

Many internal operations of fMRIPrep use Nilearn 0.8.1 (Abraham et al., 2014, RRID:SCR_001362), mostly within the functional processing workflow. For more details of the pipeline, see the section corresponding to workflows in fMRIPrep (2019) documentation. The above boilerplate text was automatically generated by fMRIPrep with the express intention that users should copy and paste this text into their manuscripts unchanged. It is released under the CC0 license.

### 2.5 Modeling

For run-level analyses, the preprocessed time series were assessed with algorithms from the Artifact Removal Toolbox (ART) to identify frames within the run that had an abnormal amount of motion (0.4 mm of total displacement, or an intensity spike >3 s.d. from the mean). The design matrix included boxcars for the experimental conditions convolved with a double-gamma hemodynamic response function (HRF), and nuisance regressors representing frame-wise motion, the anatomical CompCor regressors derived from white matter and cerebrospinal fluid, as well as impulse regressors for volumes identified by ART. A high-pass filter (120 Hz) was applied to the design matrix and the smoothed data. The model was evaluated using FSL’s FILM program. Subject-level contrast maps will be generated using FSL’s FLAME in fixed-effects mode.

### 2.6 Data exclusion

For one participant, two runs were lost due to an experimenter error that prevented recovery of event timings, resulting in six usable runs. For three participants, 3–5, runs were excluded due to excessive motion. For five participants, there was one run in which the script froze toward the end of the run. These runs were kept, and events were modeled up to the point at which the script froze. Following these exclusions, participants had either three (N = 14), two (N = 1), or one (N = 1) usable localizer run, and either eight (N = 12), six (N = 1), or five (N = 3) usable experimental runs.

### 2.7 ROI definition

ROIs included three scene-selective regions: the occipital place area (OPA), parahippocampal place area (PPA), and medial place area (MPA), as well as one early visual cortex control region (EVC). We first hand defined a contiguous cluster of scene-selective voxels for each scene region in each subject based on the contrast of scenes > objects. The localizer data were thresholded at p < 10^-3^, and only voxels surviving this threshold were included. OPA, PPA, and MPA were then defined as the top 50 voxels within the hand-defined mask in each hemisphere. Selected voxels were not required to be contiguous. All regions were defined in all participants, except for one subject missing OPA, and a second subject missing both PPA and MPA. For EVC, we used a group-constrained, subject-specific (GSS) method (Julian et al., 2012) in which we selected the top 50 voxels from each hemisphere within a larger anatomical search space taken fromWang et al. (2015). Top EVC voxels were ranked and selected based on the contrast of all conditions > fixation.

### 2.8 Univariate analyses

For univariate analyses, responses of all voxels in each ROI were averaged together for each run, and then averaged across runs, yielding the overall response of each ROI in each participant to each experimental condition. Responses were extracted separately from each hemisphere, and then averaged across hemispheres for all analyses, except where hemispheric differences were expected based on the visual field (contralateral vs. ipsilateral) of presentation (seeSection 3).

### 2.9 Multivariate analyses

For multivariate analysis, we used a split-half procedure in which the average response from each voxel was calculated across all possible combinations of half of the usable runs (thus leaving out a complementary, independent half of the runs for each fold of the data). For subjects with five usable runs, we analyzed splits of two vs. three runs. For each fold, we estimated the similarity of voxel-wise patterns of activity between conditions by calculating the Euclidean distance between each condition and every other condition (including itself) in the other half of the data. We chose to use Euclidean distance as this measure captures information in both the spatial pattern and overall strength of activation, whereas other measures (e.g., correlation distance) capture only the spatial pattern. These estimates were averaged across all folds, yielding an overall estimate of the condition-wise similarity space for each ROI and participant.

## 3 Results

### 3.1 OPA represents navigational affordances in dynamic scenes

#### 3.1.1 Multivariate analyses

If OPA represents navigational affordances in dynamic scenes, then OPA responses should differ depending on the location of navigational affordances present in the scene. To test this prediction, we analyzed conditions depicting ego-motion through scenes with either (i) an open doorway to the left and a painting on the right (conditions 2 and 5), (ii) an open doorway to the right and a painting on the left (conditions 3 and 6), or (iii) two open doorways (conditions 1 and 4). We explored responses to these conditions in a series of multivariate and univariate analyses. Conditions with turns (i.e., conditions 7–10) were not included in these analyses, since turning changes the direction of navigational affordance information during the 2.5-s trial.

For multivariate analyses, we predicted more similar responses (measured using Euclidean distance) between each of the three affordance conditions (door to the left, right, or both) and itself (across split halves of the data; “within” conditions) than between each affordance condition and the others (also calculated across split halves of the data; “between” conditions). Full hypothesis matrices are shown inFigure 3A. Further, we predicted stronger decoding of navigational affordance information in OPA than in PPA and MPA. We first tested this prediction using all possible pairs of conditions (i.e., the “overall” matrix inFig. 3A). Indeed, paired samples t-tests (one tailed) revealed significant navigational affordance decoding in OPA (t_(14)_= 3.42, p = 0.002), a smaller but significant effect in PPA (t_(14)_= 2.15, p = 0.03), and no significant effect in MPA (t_(14)_= 1.28, p = 0.11) (Fig. 3B). Directly comparing between regions, a 3 (region: OPA, PPA, MPA) x 2 (affordance: within, between) repeated measures ANOVA revealed a significant region x affordance interaction (F_(1.22,15.85)_= 5.65, p = 0.03), driven by stronger responses to affordance information in OPA than in both PPA (post hoc 2 x 2 interaction contrast; F_(1,13)_= 5.76, p = 0.03) and MPA (post hoc 2 x 2 interaction contrast; F_(1,13)_= 6.21, p = 0.03). PPA and MPA did not significantly differ (post hoc 2 x 2 interaction contrast; F_(1,14)_= 1.48, p = 0.24). Next, to provide a stronger test of navigational affordance representation, we tested for representation of navigational affordances generalizing over ego-motion information (moving forward vs. moving backward) by limiting comparisons to pairs of conditions that differed in ego-motion information, thereby minimizing the lower level visual similarity of the “within”-condition comparisons. Paired samples t-tests (one tailed) again revealed significant navigational affordance decoding in OPA (t_(14)_= 3.45, p = 0.002), a smaller but significant effect in PPA (t_(14)_= 2.80, p = 0.007), and no significant effect in MPA (t_(14)_= 0.22, p = 0.42) (Fig. 3B). Directly comparing between regions, a 3 (region: OPA, PPA, MPA) x 2 (affordance: within, between) repeated measures ANOVA revealed a significant region x affordance interaction (F_(2,26)_= 6.67, p < 0.001), driven by stronger responses to affordance information in OPA than in both PPA (post hoc 2 x 2 interaction contrast; F_(1,13)_= 6.64, p = 0.02) and MPA (post hoc 2 x 2 interaction contrast; F_(1,13)_= 8.21, p = 0.01).

#### 3.1.2 Univariate analyses

**Fig. 4.**
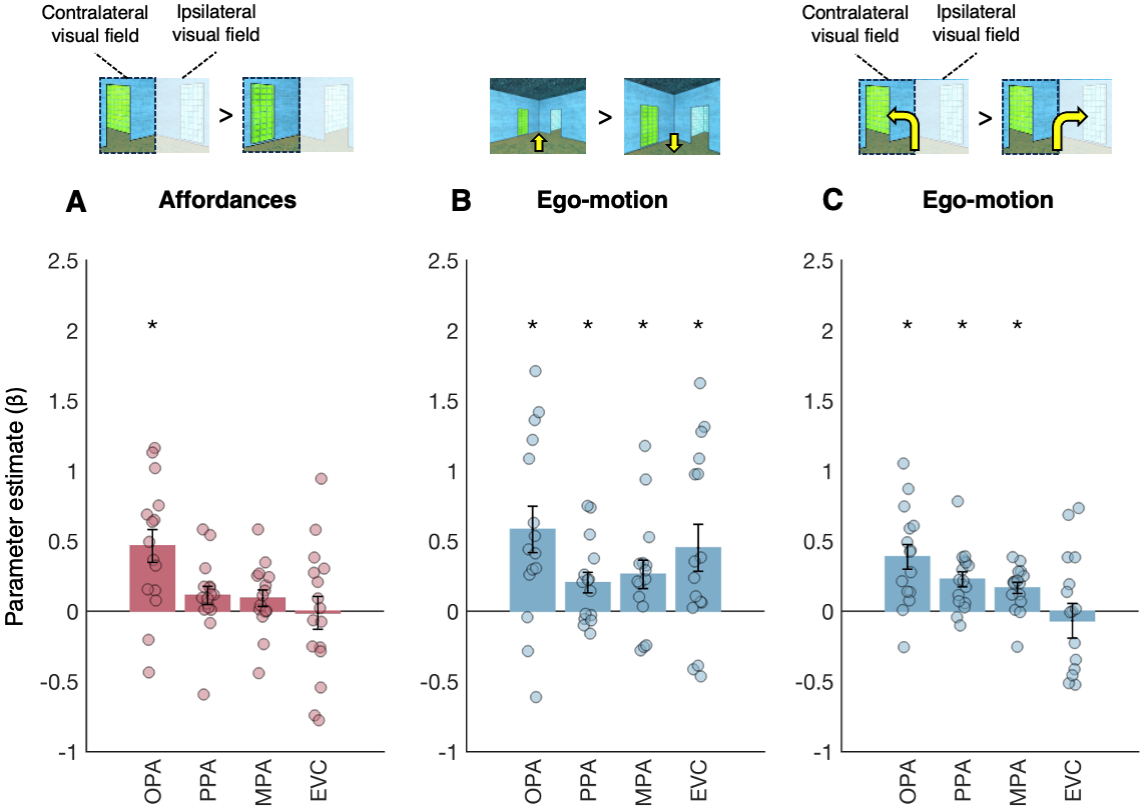
Univariate analyses of navigational affordance and ego-motion representation. (A) Navigational affordance representation was tested based on the visual field (contralateral vs. ipsilateral) in which the doorway was presented. If OPA represents navigational affordances, then stronger responses will be observed when the contralateral visual field is presented with a doorway vs. a painting. Bar plots indicate the difference in response to doorways minus paintings. (B) Forward vs. backward ego-motion information was tested using the whole visual field. If OPA represents ego-motion directions, then different responses will be observed for forward vs. backward motion. Bar plots indicate the difference in response to forward minus backward motion. (C) Turning direction information (e.g., to the left or right) was also tested based on the visual field (contralateral vs. ipsilateral) of presentation. If OPA represents ego-motion turning, then OPA responses will depend on turn direction, with stronger responses for turns toward the contralateral than toward the ipsilateral visual field. Bar plots indicate the difference in response to contralateral minus ipsilateral turns. For all plots, error bars indicate the standard error of the mean, and markers indicate data from individual participants. Asterisks indicate p < 0.05, based on paired samples t tests.

In univariate analyses, we tested the prediction that OPA will respond more to scene features that afford navigation (i.e., open doorways) vs. those that do not (i.e., paintings). To test this prediction, we focused on single-door scene conditions with either (i) an open doorway to the left and a painting on the right (conditions 2 and 5) or (ii) an open doorway to the right and a painting to the left (conditions 3 and 6). Given previous evidence that OPA shows a strong contralateral visual field bias (MacEvoy & Epstein, 2007;Silson et al., 2015,^2022^), we predicted that OPA in each hemisphere would respond more to doors than to paintings in the contralateral visual field (i.e., right OPA responding more to doors in the left than the right visual field, and vice versa for left OPA). To maximize statistical power, responses to contralateral doors vs. paintings were averaged across hemispheres, and then compared using a paired samples*t*-test (one-tailed). We found stronger responses to contralateral doors than paintings in OPA (t_(14)_= 3.84, p < 0.001), but not in PPA (t_(14)_= 1.73, p = 0.05) or MPA (t_(14)_= 1.57, p = 0.07) (Fig. 4A). Directly comparing between regions, a 3 (region: OPA, PPA, MPA) x 2 (affordance: contralateral, ipsilateral) repeated measures ANOVA revealed a significant region x affordance interaction (F_(1.13,15.85)_= 8.73, p = 0.008), with a stronger contralateral affordance preference in OPA than in both PPA (post hoc 2 x 2 interaction contrast; F_(1,14)_= 10.10, p = 0.007) and MPA (post hoc 2 x 2 interaction contrast; F_(1,14)_= 8.40, p = 0.01). PPA and MPA did not significantly differ (post hoc 2 x 2 interaction contrast; F_(1,15)_= 0.27, p = 6.13).

### 3.2 OPA responses to navigational affordances are dissociable from those in EVC

Open doorways potentially have greater motion energy than paintings, due to changing visual features seen through the doorway as the viewer moves through the scene. To better understand the role of low-level, retinotopic visual properties in driving OPA responses, we next compared responses in OPA with those in EVC. For multivariate analyses, we first tested overall navigational affordance decoding, and failed to find a significant effect in EVC (t_(15)_= 1.63, p = 0.06) (Fig. 3B). Directly comparing EVC with OPA, a 2 (affordance: within, between) x 2 (region: OPA, EVC) repeated measures ANOVA failed to reveal a significant affordance x region interaction (F_(1,14)_= 1.10, p = 0.31). We next tested navigational affordance decoding when generalizing across ego-motion directions (i.e., forward vs. backward ego-motion), minimizing the influence of low-level visual features. In this case, navigational affordance decoding in EVC was again not significant (t_(15)_= 1.62, p = 0.06), and a 2 (affordance: within, between) x 2 (region: OPA, EVC) repeated measures ANOVA failed to reveal a significant affordance x region interaction (F_(1,14)_= 2.20, p = 0.16). Accordingly, multivariate analyses were inconclusive; although significant decoding of navigational affordances could not be detected in EVC, the multivariate responses in this region did not differ from OPA upon direct comparison. To gain further clarity on this issue, we next performed univariate analyses of responses to contralateral doors vs. paintings. In this case, and unlike OPA, greater responses to contralateral doors than paintings were not detected in EVC (t_(15)_= -0.10, p = 0.54). A 2 (region: OPA, EVC) x 2 (affordance: contralateral, ipsilateral) repeated measures ANOVA revealed a significant region x affordance interaction (F_(1,14)_= 7.31, p = 0.02), with stronger responses to contralateral affordance information in OPA than EVC (Fig. 4A).

**Fig. 5.**
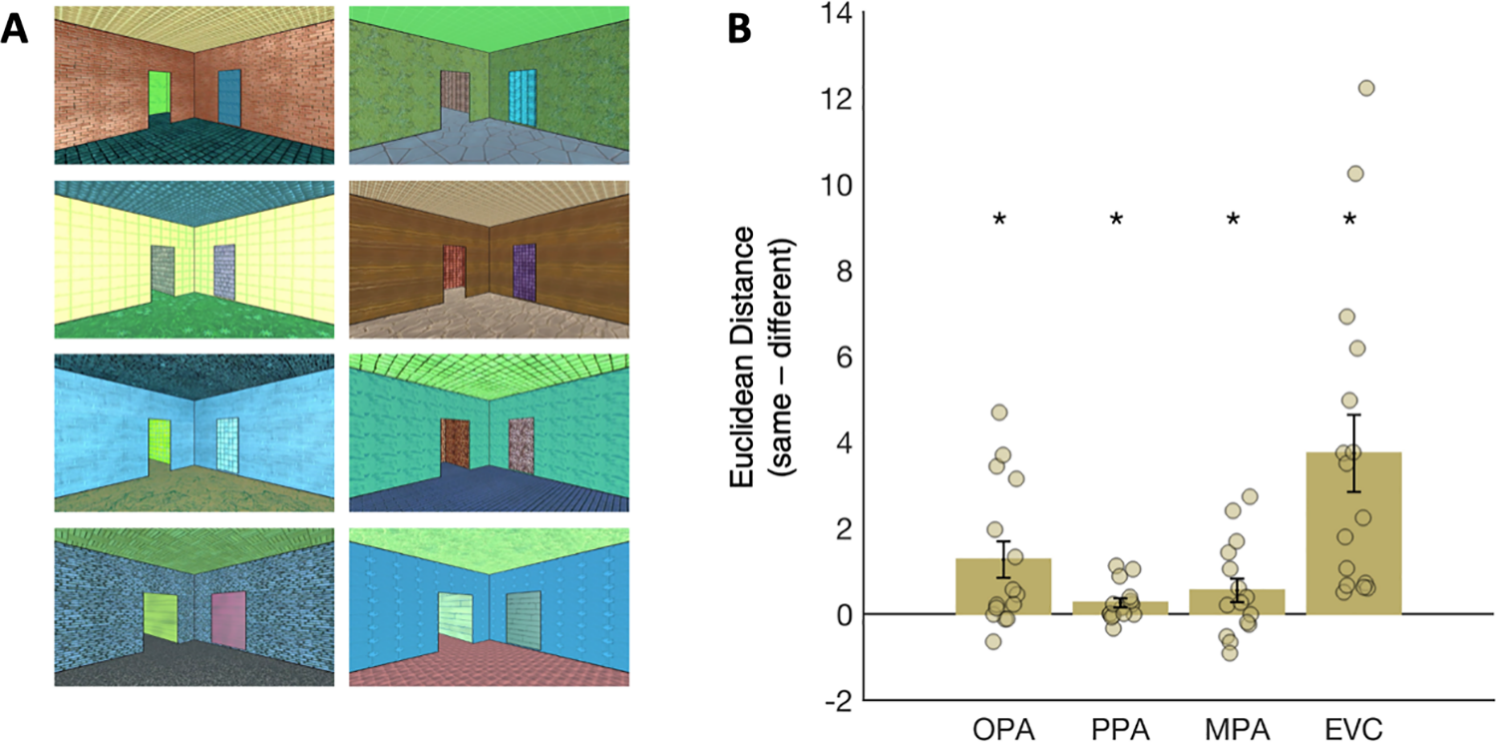
Multivariate analysis of room texture representation. To determine whether OPA responses are dissociable from those in EVC, we tested for information about room textures in each region. (A) Subjects viewed eight different room types (counterbalanced across conditions), which differed based on the textures and colors applied to the walls, floors, and ceiling. If a region is sensitive to lower-level texture information, then voxel-wise patterns of activity will be more similar (Euclidean distance) for stimuli with the same room texture than with different room textures. (B) Bar plots indicate the difference score for the same minus the different room type comparisons; responses greater than zero, therefore, indicate more similar responses (i.e., smaller Euclidean distance) for the “same” than for “different” pairs, and thus significant decoding. Error bars represent the standard error of the mean. Markers indicate data from individual participants. Asterisks indicate p < 0.05, based on paired samples t tests.

The univariate results above provide evidence of a single dissociation in responses between OPA and EVC, with greater sensitivity to navigational affordances in OPA than in EVC. However, stronger evidence that representations in these regions differ would require testing whether the opposite dissociation can also be found; that is, whether there is some information represented more strongly in EVC than in OPA. To test this possibility, we considered room texture information. Subjects viewed 8 different room types (counterbalanced across the 10 experimental conditions), which differed based on the textures and colors applied to the walls, floors, and ceiling (i.e., lower-level visual features potentially represented in EVC;Fig. 5A). If a region is sensitive to lower-level texture information, then voxel-wise patterns of activity will be more similar for stimuli with the same room texture than with different room textures. Results from this multivariate analysis showed significant decoding in EVC as well as all three scene regions (EVC: t_(15)_= 4.15, p < 0.001; OPA: t_(14)_= 2.90, p = 0.006; PPA: t_(14)_= 2.34, p = 0.02; MPA: t_(14)_= 1.93, p = 0.04) (Fig. 5B). However, the strength of this effect differed between regions, with significantly stronger sensitivity to room texture information in EVC than in OPA (repeated measures ANOVA, region x room type interaction: F_(1,14)_= 7.49, p = 0.02), as well as in PPA (region x room type interaction: F_(1,14)_= 13.54, p = 0.002) and in MPA (region x room type interaction: F_(1,14)_= 13.12, p = 0.003).

### 3.3 OPA represents ego-motion in dynamic scenes

#### 3.3.1 Multivariate analyses

If OPA represents ego-motion through dynamic scenes, then OPA responses will differ depending on the direction of ego-motion through the scene. To test this prediction, we analyzed conditions depicting forward (conditions 1–3), backward (conditions (4–6), left turn (conditions 7 and 8), and right turn (conditions 9 and 10) ego-motion. As above, we explored responses to these conditions in a series of multivariate and univariate analyses.

For multivariate analyses, we predicted more similar responses (i.e., smaller Euclidean distances) between each of the four ego-motion directions (forward, backward, left, or right) and itself (across split halves of the data) than between different pairs of conditions (also calculated across split halves of the data). Full hypothesis matrices are shown inFigure 3(top row). Importantly, to minimize visual confounds, between-condition comparisons were limited to the most closely matched pairs of conditions: left vs. right turn conditions (which were exactly the same stimuli, but mirror flipped) and forward vs. backward conditions (which were exactly the same stimuli, but temporally reversed). Paired samples t-tests revealed significant ego-motion decoding in OPA (t_(14)_= 2.43, p = 0.01), as well as in PPA (t_(14)_= 2.71, p = 0.008) and in MPA (t_(14)_= 2.25, p = 0.02) (Fig. 3B). Directly comparing between regions, a 3 (region: OPA, PPA, MPA) x 2 (ego-motion decoding: within, between) repeated measures ANOVA failed to reveal a significant interaction between region and ego-motion (F_(2,26)_= 1.16, p = 0.33). To provide an even stronger test of ego-motion representation, we also tested ego-motion representation now generalizing across conditions that differed in navigational affordance information. Paired samples t-tests revealed significant ego-motion decoding in OPA (t_(14)_= 1.99, p = 0.03) and PPA (t_(14)_= 2.41, p = 0.02), but not in MPA (t_(14)_= 1.57, p = 0.07) (Fig. 3B). However, directly comparing between regions, a 3 (region: OPA, PPA, MPA) x 2 (ego-motion decoding: within, between) repeated measures ANOVA failed to reveal a significant interaction between region and ego-motion (F_(2,26)_= 2.18, p = 0.13).

#### 3.3.2 Univariate analyses

To further explore ego-motion representation, we also performed two univariate analyses. Our first analysis tested the prediction that OPA would respond differently to forward motion (conditions 1–3) vs. backward motion (conditions 4–6), tested with a paired-samples t-test (two-tailed). Indeed, we found a significantly greater response to forward motion than backward motion in OPA, as well as in PPA and MPA (all t’s > 2.52, all p’s < 0.03) (Fig. 4B). Direct comparison between regions revealed a functional dissociation: a 3 (region: OPA, PPA, MPA) x 2 (ego-motion: forward, backward) repeated measures ANOVA revealed a significant region by ego-motion interaction (F_(2,26)_= 6.22, p = 0.006), driven by a stronger forward ego-motion preference in OPA than in both PPA (post hoc 2 x 2 interaction contrast; F_(1,13)_= 8.92, p = 0.01) and MPA (post hoc 2 x 2 interaction contrast; F_(1,13)_= 5.36, p = 0.04). There was no significant difference between PPA and MPA (post hoc 2 x 2 interaction contrast; F_(1,14)_= 0.55, p = 0.47)

Our second univariate analysis tested the prediction that responses to ego-motion directions differ by the visual field of presentation, with stronger responses to turns toward the contralateral than toward ipsilateral visual field (i.e., right OPA responding more to left turns than to right turns, and vice versa for left OPA). Note that turns toward the contralateral visual field generate greater motion energy in the ipsilateral field. Accordingly, if a region responds more to contralateral than to ipsilateral turns, it is unlikely that this effect is driven by motion energy. To maximize statistical power, responses to contra- and ipsilateral turns were averaged across hemispheres and compared using a paired-samples*t*-test (one-tailed). We found stronger responses to contralateral than to ipsilateral turns in OPA (t_(14)_= 4.27, p < 0.001), as well as in PPA (t_(15)_= 4.22, p < 0.001) and MPA (t_(15)_= 4.21, p < 0.001) (Fig. 4C). Directly comparing between regions, a 3 (region: OPA, PPA, MPA) x 2 (turn: contralateral, ipsilateral) repeated measures ANOVA did not reveal a significant region by ego-motion interaction (F_(2,26)_= 2.70, p = 0.09).

### 3.4 OPA responses to ego-motion are dissociable from those in EVC

We next compared ego-motion representation in OPA with that in EVC. For multivariate analyses, we first tested overall ego-motion decoding, and found significant sensitivity to ego-motion direction in EVC (t_(15)_= 2.94, p = 0.005), with no significant difference in the strength of ego-motion decoding between OPA and EVC; a 2 (region: OPA, EVC) x 2 (ego-motion decoding: within, between) repeated measures ANOVA failed to find a significant ego-motion by region interaction (F_(1,14)_= 0.03, p = 0.88) (Fig. 3B). Next, we tested ego-motion direction information generalizing across navigational affordance information, providing a stronger test of ego-motion representation over and above low-level visual features. In this analysis, decoding of ego-motion direction was no longer significant in EVC (t_(15)_= 0.80, p = 0.22), although direct comparison of OPA and EVC using a 2 (region: OPA, EVC) x 2 (ego-motion decoding: within, between) repeated measures ANOVA failed to reveal a significant region by ego-motion interaction (F_(1,14)_= 2.30, p = 0.15) (Fig. 3B). Finally, we performed two univariate analyses.

First, for univariate analyses of forward vs. backward motion responses, we found a preference for forward over backward motion in EVC (paired samples t-test, t_(15)_= 2.70, p = 0.02), with no difference in response between OPA and EVC (region by ego-motion interaction; F_(1,14)_= 0.42, p = 0.53) (Fig. 4B). Second, for univariate analyses comparing contralateral vs. ipsilateral turns, we found no significant difference in the response to contra- than to ipsilaterally presented turns in EVC (t_(15)_= 0.55, p = 0.70), and a 2 (region: OPA, EVC) x 2 (turn: contralateral, ipsilateral) repeated measures ANOVA revealed a significant region x turn interaction (F_(1,14)_= 10.95, p = 0.005) (Fig. 4C). Taken together, these results suggest that OPA representation of ego-motion may be partially, but not entirely explained by low-level, retinotopic visual information. Indeed, although EVC responses can discriminate between basic ego-motion directions, the full profile of OPA responses to ego-motion is not detectable in EVC—particularly for tests of ego-motion representation that minimize the influence of low-level visual features (e.g., when generalizing across different navigational affordance conditions, or for contralateral vs. ipsilateral turns).

### 3.5 Integration of navigational affordances and ego-motion

**Fig. 6.**
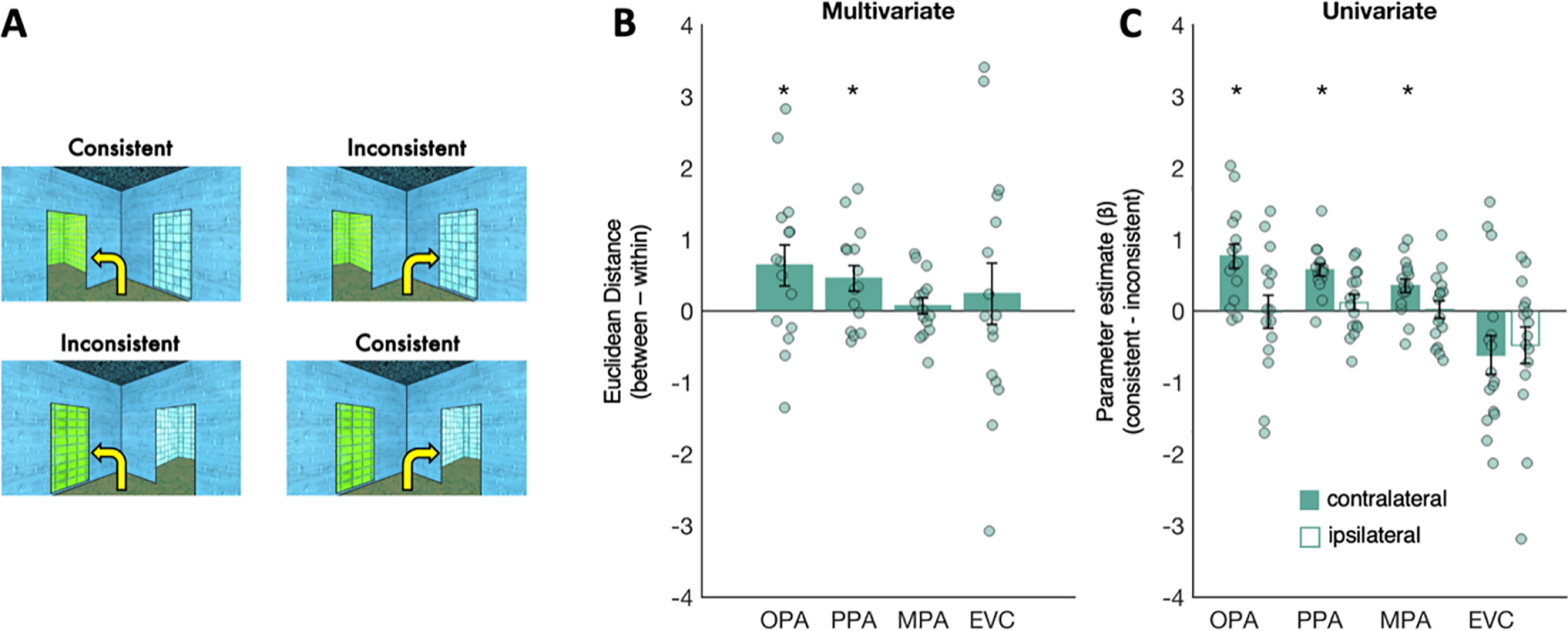
Testing the conflict of navigational affordance and ego-motion information. (A) Conditions used for conflict analyses. Navigational affordance and ego-motion are consistent when turns are taken in the direction of the open doorway (top left and bottom right conditions). This information is put in conflict when turns are taken in the opposite direction of the doorway (top right and bottom left conditions). (B) Multivariate analyses. If OPA represents the conflict of navigational affordance and ego-motion information, then more similar responses will be observed for conditions with the same conflict information (consistent–consistent, inconsistent–inconsistent) than the different conflict information (consistent–inconsistent). Bar plots indicate the difference score for the “between” conditions minus the “within” conditions; responses greater than zero, therefore, indicate more similar responses (i.e., smaller Euclidean distance) for the “within”- than for “between”-condition pairs, and thus significant decoding. (C) Univariate analyses were conducted with respect to the visual field of presentation. If OPA represents the conflict of navigational affordance and ego-motion information, specifically in the contralateral visual field, then stronger responses to consistent than to inconsistent turns will be found for contralateral turns, but not to ipsilateral turns. Bar plots indicate the difference in response to consistent minus inconsistent turns. For all plots, error bars represent the standard error of the mean. Markers indicate data from individual participants. Asterisks indicate p < 0.05, based on paired samples t tests.

The analyses above suggest that OPA plays a unique role in the scene network by representing both navigational affordances and ego-motion in dynamic scenes. Does OPA integrate these two kinds of navigationally relevant information? To begin to address this question, we compared responses with conditions in which navigational affordance and ego-motion information are consistent (e.g., an open doorway to the left, with a turn toward the door; conditions 7 and 10) vs. inconsistent (e.g., an open doorway to the left, with a turn away from the door; conditions 8 and 9) (Fig. 6A). Once again, we tested responses to these conditions with a series of multivariate and univariate analyses.

For multivariate analyses, we predicted more similar responses within consistent and inconsistent conditions (across split halves of the data) than between these conditions (also calculated across split halves of the data) (Fig. 6B). Paired samples t-tests revealed a significant effect in OPA (t_(14)_= 2.20, p = 0.02) and PPA (t_(14)_= 2.56, p = 0.01), but not in MPA (t_(14)_= 0.65, p = 0.26). A 3 (region: OPA, PPA, MPA) x 2 (decoding: within, between) repeated measures ANOVA did not find a significant region x decoding interaction (F_(1.26,16.4)_= 1.96, p = 0.18). Notably, the differential response to consistent vs. inconsistent turning conditions was not likely explained by low-level, retinotopic visual features, since no significant effect was found in EVC (paired samples t-test, t_(15)_= 0.56, p = 0.29), although direct comparison between EVC and OPA did not reveal a significant region x decoding interaction (F_(1,14)_= 0.44, p = 0.52).

To further explore how navigational affordance and ego-motion information are integrated, we next tested the univariate prediction that OPA would respond differently to consistent vs. inconsistent turns, particularly when this information is presented in the contralateral visual field (e.g., for right OPA, a preference for left turns toward an open doorway vs. left turns toward a painting). Consistent with this prediction, we found greater OPA responses to consistent than to inconsistent turns toward the contralateral visual field (paired samples t-test, t_(14)_= 4.48, p < 0.001). This effect was not likely explained by a simple preference for doors vs. paintings in the contralateral visual field, since no difference in response was found for consistent vs. inconsistent ipsilateral turns (paired samples t-test, t_(14)_= 0.05, p = 0.52); if OPA is simply responding to doorways in the contralateral visual field, then OPA would respond significantly more to inconsistent, ipsilateral turns (which contain a doorway in the contralateral visual field) than to consistent, ipsilateral turns (which contain a painting in the contralateral visual field). The preference for consistent contralateral turns, but not ipsilateral turns, was also observed in PPA and MPA (paired samples t-tests; contralateral: both t’s_(15)_> 3.68, both p’s < 0.002; ipsilateral: both t’s_(15)_= 1.06, p = 0.15). To directly compare between regions, we first calculated the difference in response to the consistent minus the inconsistent condition in each visual field, and then submitted these values to a 2 (visual field: contralateral, ipsilateral) x 3 (region: OPA, PPA, MPA) repeated measures ANOVA. This analysis failed to reveal a significant interaction between visual field and region (F_(2,28)_= 2.70, p = 0.09). In contrast to the scene regions, EVC responded more to inconsistent than to consistent turns in the contralateral visual field (paired samples t-test, t_(15)_= 2.27, p = 0.04), with no significant difference in response to inconsistent than to consistent turns in the ipsilateral visual field (paired samples t-test, t_(15)_= 1.88, p = 0.08). In sum, the scene regions, and particularly OPA, integrate navigational affordance and ego-motion information, responding maximally when they converge.

## 4 Discussion

Here we used dynamic movie stimuli to test how navigationally relevant information is represented in scene-selective cortex during the first-person visual experience of navigating scenes. We found that one scene region, the OPA, was sensitive to both navigational affordances (i.e., the presence vs. absence of open doorways affording further navigation) and ego-motion directions (i.e., walking forward vs. backward, turning left vs. right). Results were less consistent in two other scene regions, the PPA and MPA, which showed weaker or no sensitivity to navigational affordance information, and relatively weaker evidence of ego-motion representation. These results support the hypothesis that regions of the cortical scene processing system are functionally dissociated, with OPA playing a specific role in representing navigationally relevant information in dynamic visual scenes.

Our results reveal a more detailed functional profile for OPA than previously known. Past work has shown that OPA is activated more strongly by dynamic than by static scenes (Hacialihafiz & Bartels, 2015;Kamps et al., 2016b,Kamps et al., 2020;Sulpizio et al., 2020;Suzuki, 2021), and shows a preference for dynamic scenes viewed from a walking perspective, rather than a crawling or flying perspective (Jones et al., 2023;Jung et al., 2024). Here we explored the more specific representations in OPA during the visual experience of navigating through a scene, revealing that OPA represents information about both the structure of the space as it constrains possibilities for future navigation (i.e., navigational affordances) and the dynamics of ongoing motion through the space (i.e., ego-motion), and their interaction. Given that navigational affordance information is particularly useful for understanding possibilities for future actions (e.g., which way can I walk next?) while ego-motion reveals the current trajectory of motion (e.g., how am I currently moving?), these results suggest that OPA is involved not only in navigational planning, but also in online guidance of navigation.

Prior work has typically studied (static) scene perception and ego-motion perception separately, with studies of static scene perception focused on the three regions tested here (OPA, PPA, and MPA), and studies of ego-motion focused on a network of regions in dorsal visual cortex, parietal cortex, premotor cortex, and the cerebellum (e.g.,Di Marco, et al., 2021;Pitzalis et al., 2020;Ruehl et al., 2022;Sulpizio et al., 2020,^2023^;Wall & Smith, 2008). The current results support the idea that OPA may play a relatively unique role at the intersection of these two networks (Sulpizio et al., 2020), by representing information about navigational affordances—a property of static scene geometry—and ego-motion direction—a property only defined in dynamic scenes. We speculate that the co-localization of these two types of information in OPA might support navigation by facilitating comparison between planned navigational trajectories and currently observed progress in following that trajectory. Supporting this idea, we found initial evidence that OPA is sensitive to the conflict of navigational affordance and ego-motion information (e.g., turning toward vs. away from an open doorway)—although this effect was not specific to OPA, but also found in other scene regions.

Although OPA is sensitive to higher-level information relevant to navigation, OPA responses are nevertheless constrained by the visual field of presentation. Mirroring the broader organization of early visual processing, past studies have demonstrated a clear preference in OPA for contralateral visual stimulation, particularly in the upper visual field (MacEvoy & Epstein, 2007;Silson et al., 2015,^2022^).Silson et al. (2015)further showed that visual field biases in OPA (and other scene regions) are stronger when mapped with fragments of static scene images, compared with simple checkerboard stimuli, suggesting that OPA encodes high-level representations of scene stimuli, particularly in the lower, contralateral visual field.Bonner and Epstein (2018)further used computational modeling to suggest that navigationally relevant scene information is typically present in the lower visual field. Our results build on this finding by revealing the more specific scene information extracted by OPA in this portion of the visual field, namely, navigational affordances and ego-motion. Future work will be required to explore how the navigationally relevant information encoded separately in each hemisphere is integrated to form a coherent representation across the entire visual field, for example, facilitating decision making about which paths to follow to the left vs. right.

Notably, OPA is adjacent to, and overlapping with area V3A an ego-motion sensitive region, raising the possibility that ego-motion responses could be attributable to this region instead. The relationship between V3A and OPA is not yet well understood. One possibility is that these two regions, generally studied separately and localized using different contrasts, are actually the same region. Consistent with this idea, area V3A has been shown to respond more to scenes than faces and overlaps heavily with OPA (Sulpizio et al, 2020). An alternative hypothesis is that these regions are functionally distinct, but functional differences between these regions are challenging to detect due to their proximity, relative to the spatial resolution of fMRI. Clearly then, more work is needed to understand whether and how visual representations in these regions differ.

While ego-motion representation was found in OPA, at least some sensitivity to this information was found in all other regions tested, including EVC. Both OPA and EVC further showed a preference for forward (vs. backward) ego-motion, suggesting that ego-motion representation is biased toward the direction most commonly experienced during visually guided navigation, and consistent with prior work showing a preference for forward vs. backward radial optic flow in ego-motion sensitive brain regions (e.g.,Di Marco et al., 2021). Widespread representation of ego-motion across the visual cortex is perhaps not surprising, given that ego-motion directions generate distinct patterns of optic flow across the visual field (e.g., a left turn causes higher motion energy in the right visual field, and vice versa for a right turn). Nevertheless, some evidence indicates that nature of ego-motion representation differs between OPA and EVC. For example, ego-motion decoding remained significant in OPA, but not in EVC, when generalizing across stimuli that differed in navigational affordance information (a test which minimizes the influence of lower-level motion energy in driving decoding performance). Moreover, univariate responses in OPA were greater for turns in the contralateral direction (e.g., left OPA responding more to right turns than to left turns). If responses in OPA were driven only by lower-level motion energy, then greater responses should be found for ipsilateral turns, which generate greater motion energy in contralateral visual field. Based on these results, we speculate that OPA encodes relatively higher-level representations of ego-motion, perhaps reflecting ongoing navigational goals, while ego-motion representation in EVC is driven more directly by low-level motion energy information (despite failing to detect greater EVC responses to ipsilateral than to contralateral turns in the current analyses).

## 5 Conclusion

In sum, we used dynamic visual stimuli to find evidence of a functional dissociation in the human cortical scene network, with OPA showing stronger and more consistent evidence of navigationally relevant information processing in dynamic scenes than PPA or MPA. Moreover, the pattern of responses in OPA could not be explained by low-level, retinotopic visual features. These results support the hypothesis that OPA plays a unique role in the cortical scene processing network by representing scenes as dynamic, navigable spaces.

## Supplementary Material

Supplementary Material

## Data Availability

The methods and analyses of this study were preregistered prior to data analysis. Deviations from the preregistered analyses, and results from originally planned analyses, are detailed in the supplemental materials. Preregistration documents, experiment scripts, stimuli, data, and analysis scripts required to produce statistical results are available athttps://osf.io/6yehp/?view_only=53ba7725d61343a29a2e3e6da5d75f28. Defaced brain images from participants who consented to share them are available athttps://doi.org/10.18112/openneuro.ds005374.v1.0.1.
