# Supplementary material for: Representation of navigational affordances and ego-motion in the occipital place area
Source: Imaging Neurosci (Camb). 2025 Jan 10;3:imag_a_00424. doi: 10.1162/imag_a_00424 (PMC12319941; doi:10.1162/imag_a_00424)
Supplement: Supplementary Material [file imag_a_00424-supp.pdf]

Supplemental materials

Deviations from preregistered analyses

Our primary analyses (as reported in the manuscript) deviated from our preregistered analyses in three ways. First, many of our preregistered analyses depended on comparisons between single pairs of conditions. After seeing the results of the preregistered analyses, we decided to perform the analyses reported in the main results of the manuscript, which involved averaging responses across a greater number of conditions, in order to increase power to detect key effects. Second, we initially planned to define scene ROIs using a group-constrained, subject specific (GSS) method, but after visually inspecting the localizer data, we discovered that the peak OPA activation often fell outside the parcel search space. To ensure accurate ROI definition, we therefore opted to define the scene ROIs by hand, as described in the manuscript. Third, and finally, we analyzed data expressed as raw betas, rather than percent signal change. For transparency, results of all original, pre-registered analyses not reported in the main results are presented below. Notably, we still opted to report these analyses in hand-defined ROIs, rather than GSS ROIs, since the GSS ROIs were determined to be inaccurate. Results are also reported as raw betas to facilitate comparison to the primary analyses. In our view, these deviations from the preregistered analysis plan are minor and well-justified. Nevertheless, because we made these changes after conducting some initial analyses, the results reported here must be considered exploratory, and should be replicated in a fully independent sample.

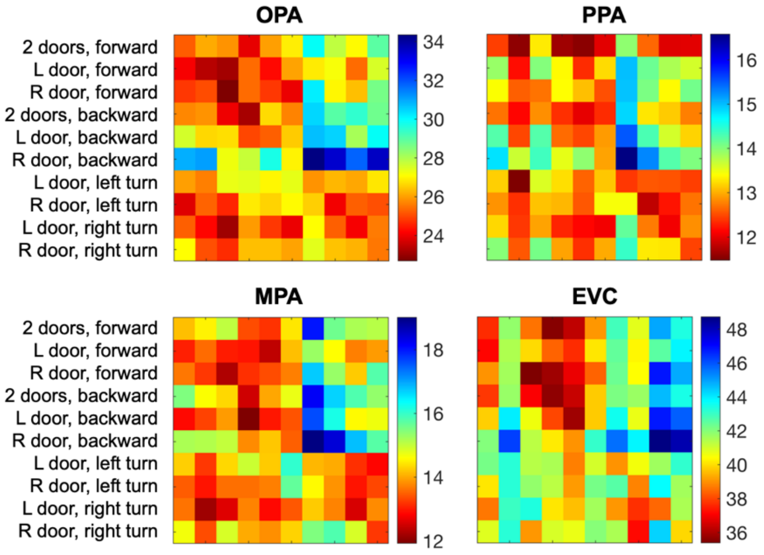

Supplemental Figure 1. Representational similarity matrices in each region. Each cell depicts the Euclidean distance between a pair of conditions, calculated across split halves of runs. The order of conditions of the y and x axis in each plot are the same.

Additional preregistered analyses of navigational affordance representation

If OPA represents navigational affordances, then OPA responses will differ depending on the number and direction of navigational affordances present in the scene. To test this prediction, we analyzed conditions 1, 2, and 3 only, which depict forward motion through scenes with either i) an open doorway to the left (condition 2); ii) an open doorway to the right (condition 3); or iii) two open doorways (condition 1). For univariate analyses, we found no evidence of differential responses to 2-door versus 1-door scenes (calculated as the average of left- and right-door scenes; two-tailed paired samples t-test) in OPA ($t_{(14)} = 1.45$, $p = 0.17$), nor in PPA ($t_{(14)} = 1.86$, $p = 0.08$), MPA ($t_{(14)} = 0.30$, $p = 0.77$), or EVC ($t_{(15)} = 1.40$, $p = 0.18$) (Supplemental Figure 2). For multivariate analyses, we predicted stronger correlations between each of the three affordance conditions (door to the left, right, or both) and itself (across split halves of the data) than between each condition and each other condition (also calculated across split halves of the data), tested with a paired samples *t*-test (one-tailed). Despite trends, we failed to find significant results in OPA ($t_{(14)} = 1.51$, $p = 0.08$), PPA ($t_{(14)} = 1.55$, $p = 0.07$), MPA ($t_{(14)} = 0.80$, $p = 0.22$) and EVC ($t_{(15)} = 1.20$, $p = 0.12$) (Supplemental Figure 2). Direct comparison between regions with repeated measures ANOVA failed to find a difference between OPA and all other regions (region x affordance interactions; all F's < 0.91, all p's > 0.37).

Positive findings in the analyses above could have been driven by a confound of lower-level dynamic visual information, since ego-motion through space will cause greater changes in the dynamic patterns of occlusion visible through the doorway, as compared with the painting. To help address this possibility, we analyzed conditions 11 and 12, which are similar to conditions 2 and 3, but depict an inverted doorway on the control side (rather than a painting) (Supplemental Figure 3). The inverted doorway is precisely matched to the open doorway in terms of lower-level dynamic visual information, except that it cannot be navigated via typical, bipedal locomotion. We made two

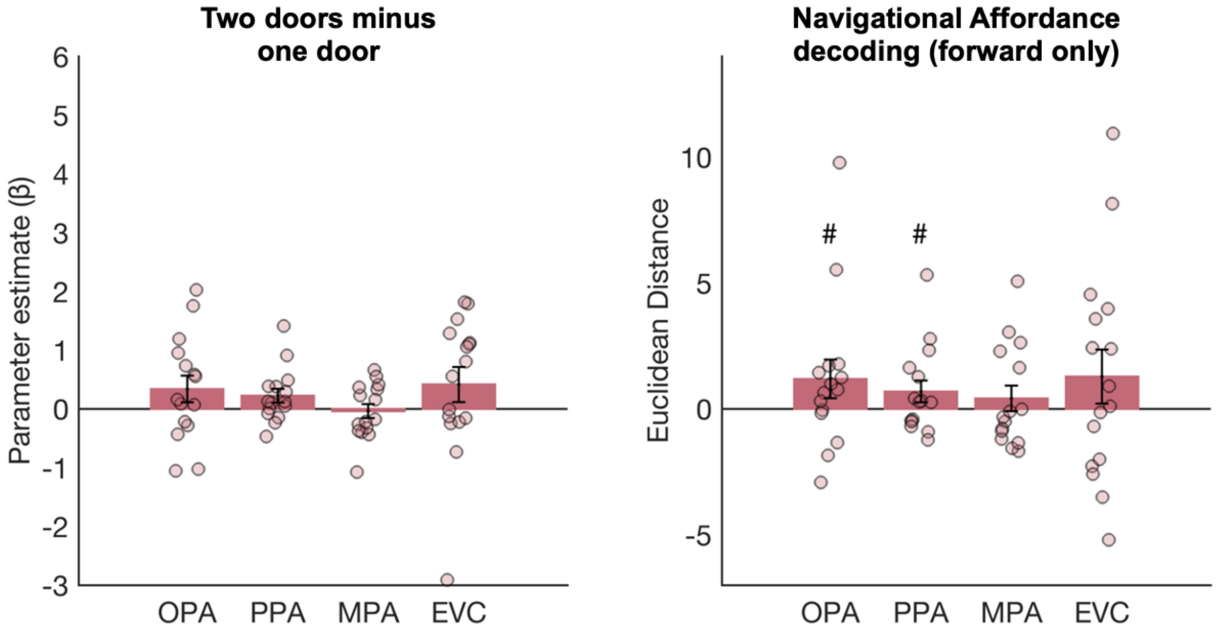

Supplemental Figure 2. *Additional tests of navigational affordance representation.* A) Univariate responses (difference scores) to two door minus one door scenes, tested using only the forward motion conditions (i.e., conditions 1-3). B) Multivariate decoding of navigational affordances tested only using forward motion conditions (i.e., conditions 1-3). For both plots, error bars represent the standard error of the mean. Markers indicate data from individual participants.

multivariate predictions. First, in order to demonstrate basic sensitivity to the direction of the navigational affordance in these conditions, we predicted that patterns of activation would be more similar between each condition and itself (across split halves of the data) than between each condition and the other condition, tested with a paired samples *t*-test (one-tailed). We failed to find a significant effect in OPA ($t_{(14)} = 1.36$, $p = 0.10$) and MPA ($t_{(14)} = 0.66$, $p = 0.26$), but did find significant effects in PPA ($t_{(14)} = 1.93$, $p = 0.04$) and EVC ($t_{(15)} = 2.83$, $p = 0.006$). The effect in OPA was significantly weaker than that in EVC (region x condition interaction, repeated measures ANOVA; $F_{(1,14)} = 5.23$, $p = 0.04$), but no differences were found between the three scene regions (region x condition interaction, repeated measures ANOVA; $F_{(1.19,15.48)} = 1.55$, $p = 0.24$). Second, we predicted that if OPA represents navigational affordances – not just low-level dynamic visual information – then coding of navigational affordance information will generalize across scene features that place similar constraints on navigation, but which differ in terms of dynamic visual information (i.e., paintings versus inverted doors). Specifically, we predicted stronger correlations across conditions 2-11 and 3-12 than across conditions 2-12 and 3-11, tested with a paired samples *t*-test (one-tailed). This analysis failed to reveal a significant effect in OPA ($t_{(14)} = 0.08$, $p = 0.53$), MPA ($t_{(14)} = 0.40$, $p = 0.65$), and EVC ($t_{(15)} = 0.75$, $p = 0.77$), although a significant effect was observed in PPA ($t_{(14)} = 1.96$, $p = 0.03$). However, no significant differences were found between the three scene regions (repeated measures ANOVA, region x condition interaction: $F_{(2,26)} = 1.32$, $p = 0.28$) or between OPA and EVC (region x condition interaction: $F_{(1,14)} = 0.66$, $p = 0.43$). Finally, to further evaluate the robustness of navigational affordance coding in OPA, we included two further inverted door conditions (conditions 13 and 14), which depicted forward motion through rooms with a different overall spatial layout than that in the conditions above (Supplemental Figure 3). If OPA represents navigational affordances, then patterns of activity should be more similar between each condition and itself (across split halves of the data) than between each condition and the other condition, tested with a paired samples *t*-test (one-tailed). Again, we failed to find a significant effect in OPA

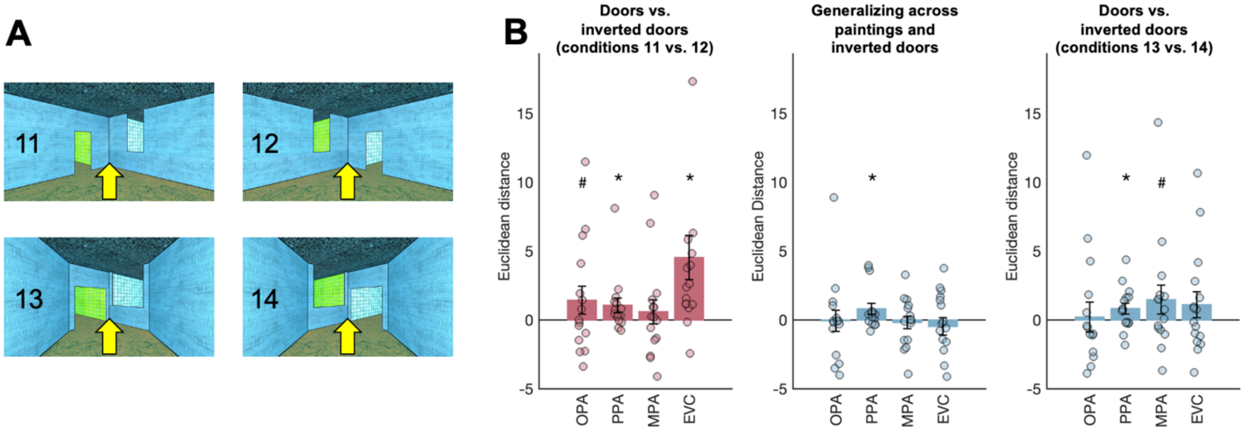

Supplemental Figure 3. *Testing responses to dooways vs. inverted doorways.* A) Example stimuli from conditions 11-14. B) Multivariate decoding results from the inverted doorway conditions. Bar plots indicate the difference score for the “between” conditions minus the “within” conditions; responses greater than zero indicate more similar responses (i.e., smaller Euclidean distance) for the “within” than “between” condition pairs, and thus significant decoding. Error bars represent the standard error of the mean. Markers indicate data from individual participants.

($t_{(14)}$ = 0.18, p = 0.43). We did observe a significant effect in PPA ($t_{(14)}$ = 2.01, p = 0.03), but not in MPA ($t_{(14)}$ = 1.36, p = 0.10) nor EVC ($t_{(14)}$ = 1.17, p = 0.13).

Additional preregistered analyses of ego-motion representation

If OPA represents navigational dynamics, then OPA responses will differ depending on the direction of ego-motion through the scene. Key pre-registered analyses testing this hypothesis are already presented in the main results. For an additional multivariate analyses, we analyzed forward (conditions 1-3) and backward (conditions 4-6) motion conditions, and predicted stronger correlations across conditions that share a direction of motion (forward-forward or backward-backward, across split halves of the data, and across all affordances) than conditions that differ in the direction of motion (i.e., forward-backward) (Supplemental Figure 4). A paired samples t-test (one-tailed) revealed a significant difference in OPA ($t_{(14)}$ = 1.99, p = 0.03) and EVC ($t_{(14)}$ = 2.40, p = 0.01), but not PPA or MPA (both t's < 1.63, both p's > 0.06). However, repeated measures ANOVAs with factors for region (OPA, PPA, and MPA; or OPA and EVC) and condition (within, between) failed to reveal significant region x condition interactions (both F's < 1.78, both p's > 0.19).

Second, we pre-registered a multivariate analysis comparing responses to scene movies depicting left turns (conditions 2 and 3) versus right turns (conditions 5 and 6). We predicted stronger correlations across conditions depicting the same direction of motion (left-left, right-right, across split halves of the data) than those that differ in the direction of motion (left-right). Paired samples t-tests (one-tailed) failed to find a significant difference in OPA ($t_{(14)}$ = 1.61, p = 0.07) and

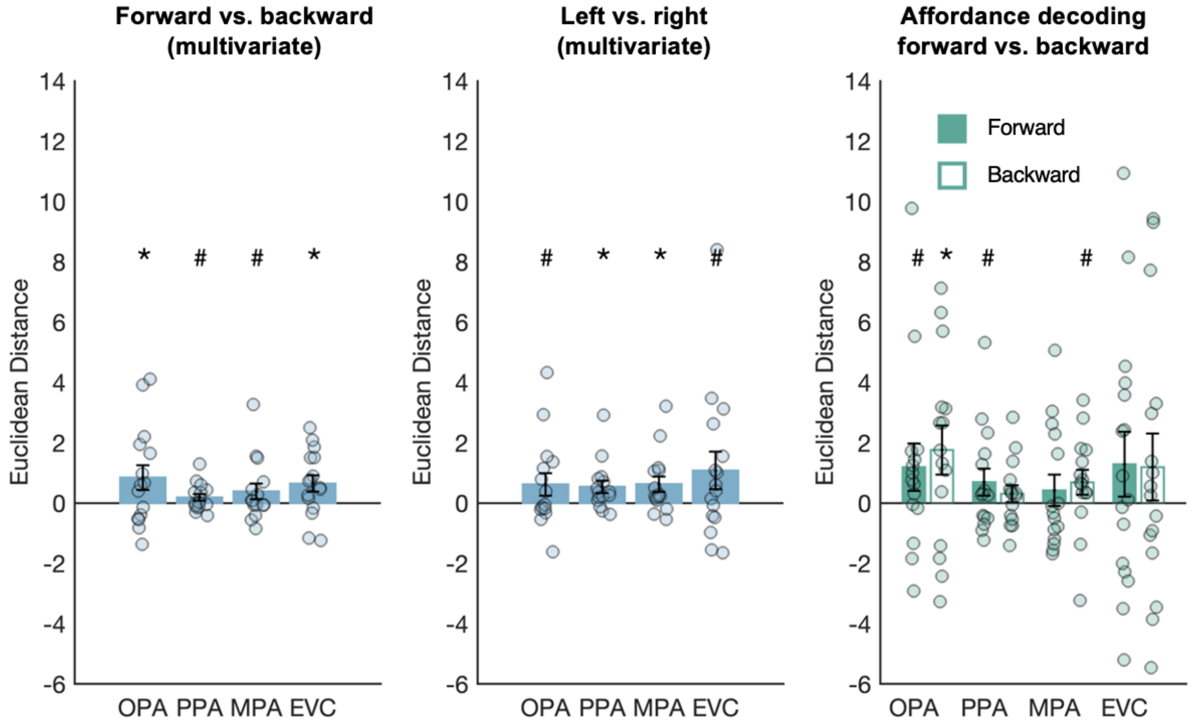

Supplemental Figure 4. *Additional tests of ego-motion representation.* Bar plots indicate the difference score for the "between" conditions minus the "within" conditions; responses greater than zero indicate more similar responses (i.e., smaller Euclidean distance) for the "within" than "between" condition pairs, and thus significant decoding. Error bars represent the standard error of the mean. Markers indicate data from individual participants.

EVC ($t_{(15)}$ = 1.74, p = 0.051), but did find significant differences in PPA ($t_{(14)}$ = 2.56, p = 0.01) and MPA ($t_{(14)}$ = 2.42, = 0.01) (Supplemental Figure 4). However, repeated measures ANOVAs with factors for region (OPA, PPA, and MPA; or OPA and EVC) and condition (within, between) failed to reveal significant region x condition interactions (both F's < 0.22, both p's > 0.70).

Finally, we asked: how are processing of navigational dynamics and structure related? Navigational dynamics might influence processing of navigational structure, since the structure of navigable space is more relevant in the direction of the current heading (e.g., ahead of you while walking forward) than in another direction (e.g., behind you while walking forward). To test this possibility, we analyzed conditions 1-6, which depicted forward or backward ego-motion through scenes with a door on either the left, right, or both walls. If navigational dynamics influence representation of navigational structure, then we will find stronger decoding of affordance direction (i.e., calculated as within- minus between-correlations for the left, right, and both conditions) for the forward conditions than the backward conditions, tested with a paired samples t-test (one tailed). We failed to find evidence of this effect in all four regions (all t's < 1.31, all p's > 0.10) (Supplemental Figure 4).

Additional preregistered analyses of the integration of navigational affordances and ego-motion

If OPA integrates navigational affordances and ego-motion then OPA responses will differ depending on whether the depicted ego-motion is consistent with the navigational affordance of the space (e.g., a left turn toward a door on the left). To test this prediction, we analyzed conditions 7, 8, 9, and 10, which depicted left or right turns in the direction of either an open doorway that affords further navigation (i.e., a "consistent" turn) or away from a doorway toward a wall containing a painting, which does not afford further navigation (i.e., an "inconsistent" turn). Key analyses testing this prediction are already present in the primary results. For an additional univariate analyses, we predicted an overall difference in response to the consistent and inconsistent conditions, tested using a two-tailed paired samples *t*-test. We found no evidence for greater response to consistent than inconsistent turns in OPA ($t_{(14)}$ = 2.07, p = 0.06), nor MPA ($t_{(14)}$ = 1.91, p = 0.08), but did find a significantly greater response to consistent than inconsistent turns in PPA ($t_{(14)}$ = 4.24, p < 0.001), and a significantly weaker response to consistent than inconsistent turns in EVC ($t_{(15)}$ = 2.36, p = 0.03). Directly comparing between the three scene regions, a 3 (region: OPA, PPA, MPA) x 2 (condition: consistent, inconsistent) repeated measures ANOVA failed to reveal a significant region x condition interaction ($F_{(1.27, 16.45)}$ = 1.39, p = 0.26). However, comparing OPA and EVC, a 2 (region: OPA, EVC) x 2 (condition: consistent, inconsistent) repeated measures ANOVA did reveal a significant region x condition interaction ($F_{(1, 14)}$ = 19.19, p < 0.001).
